# The Associations Between Temperament and Self-Oriented, Other-Oriented, and Dual-Harmful Behaviors in Emerging Adults

**DOI:** 10.5334/pb.1327

**Published:** 2024-12-30

**Authors:** Serafine Dierickx, Dirk Smits, Glenn Kiekens, Laurence Claes

**Affiliations:** 1 Faculty of Psychology and Educational Sciences, KU Leuven, Leuven, Belgium; 2 Resilient People, UCLL Research & Expertise, Leuven, Belgium; 3 Odisee University of Applied Sciences, Brussel, Belgium; 4 Department of Neurosciences, Center for Contextual Psychiatry, KU Leuven, Leuven, Belgium; 5 Department of Medical and Clinical Psychology, Tilburg University, Tilburg, The Netherlands; 6 Faculty of Medicine and Health Sciences (CAPRI), University of Antwerp, Wilrijk, Belgium; 7 Child & Youth Institute, KU Leuven, Leuven, Belgium

**Keywords:** temperament, non-suicidal self-injury, aggression, dual-harm, reinforcement sensitivity, effortful control

## Abstract

Self- and other-oriented harmful behaviors are common among emerging adults. Individuals who engage in both forms of behavior, termed dual-harm, experience more adverse outcomes in comparison to individuals who engage in either. This study examines temperamental traits, defined as reactive and regulative temperament, as transdiagnostic factors underlying engagement in self-oriented, other-oriented, and dual-harmful behaviors. These harmful behaviors are operationalized as non-suicidal self-injury (NSSI), direct aggression, and a combination of both, respectively. Participants are 669 emerging adults (69.36% women; *M_age_* = 21.48; *SD* = 2.20). The study focusses on how temperament can differentiate four different groups (i.e., a no-harm, NSSI-only, aggression-only, and a dual-harm group) in a pairwise manner. Results highlight a hyper-reactive Behavioral inhibitions system (BIS) and a hypo-reactive Behavioral activation system (BAS)-Impulsivity in individuals who engage in NSSI-only, compared to no-harm and aggression-only. Conversely, we found a hypo-reactive BIS and a hyper-reactive BAS-Impulsivity in those who report aggressive behaviors, compared to no-harm and NSSI-only. Finally, a hypo-reactive Effortful control (EC) is found in the dual-harm group, when compared to those engaging only in NSSI, and hyper-reactive BIS and BAS-Impulsivity are found in the dual-harm group, when compared to those engaging only in aggressive behaviors, or those engaging in neither behaviors. This study highlights the role of temperamental traits in differentiating patterns of self-oriented, other-oriented, and dual-harmful behaviors, which should be integrated in future research and clinical interventions.

## Introduction

Harmful behaviors encompass a range of maladaptive behaviors that can be categorized as either self-oriented (e.g., non-suicidal self-injury or NSSI) or other-oriented (e.g., physical and verbal aggressive behaviors). Different harmful behaviors are often studied independently, even though research suggests that they frequently co-occur and share common underlying mechanisms (Bresin & Hunt, 2023; O’Donnell et al., 2015). When both forms of harmful behaviors coincide, it is referred to as dual-harm (O’Donnell et al., 2015). Temperament, defined as individual differences in emotional reactivity and regulation (Rothbart et al., 2000), may influence engagement in harmful behaviors (Carver & Harmon-Jones, 2009; Chapman et al., 2006). The present study focuses on the most visible or noticeable types of self- and other-oriented harmful behaviors, being non-suicidal self-injury (NSSI) and aggressive behavior, respectively, or a combination, termed dual-harm, and seeks to identify how temperamental traits differentiate harmful behaviors groups, i.e., no-harm, NSSI-only, aggression-only, and dual-harm (NSSI and aggressive behavior), from each other in a sample of emerging adults. Pairwise comparison of these four groups will provide insight into the unique and shared mechanisms underlying different types of harmful behaviors in emerging adults, which creates opportunities to improve models of psychopathology and inform the development of transdiagnostic strategies to reduce harmful behaviors rather than studying each type of behavior in isolation.

### Harmful behaviors

NSSI refers to any socially or culturally unacceptable behavior inflicting direct and deliberate damage to an individual’s body tissue without suicidal intent (International Society for the Study of Self-Injury, 2022), such as self-cutting and self-burning. Lifetime NSSI is estimated to occur in 18% of adolescents, 13% of emerging adults, and 5% of adults (Muehlenkamp et al., 2012; Swannell et al., 2014). The age of onset of NSSI is most often situated in mid-adolescence (i.e., 14–16 years old), with a second peak during emerging adulthood (Cipriano et al., 2017; Gandhi et al., 2018). Many individuals who start engaging in NSSI during adolescence also continue to self-injure during emerging adulthood (Daukantaite et al., 2020; Kiekens et al., 2022). Across studies, women report slightly higher rates of NSSI than men, but the difference is usually smaller in community samples compared to clinical samples (Bresin & Schoenleber, 2015).

Aggressive behaviors can be physical (e.g., hitting someone) or verbal (e.g., threatening or yelling at someone) and refer to other-oriented types of harm. Aggressive behavior is characterized by actions intended to cause physical, psychological, or social harm to others (Lange et al., 2005). Aggressive behaviors peak between the ages 20–30 years old (Archer, 2004). Research on sex differences in aggressive behaviors shows that men express more physically aggressive behavior than women, whereas no significant sex differences are found for verbally aggressive behavior (Archer, 2004; Björkqvist, 2018).

Finally, dual-harm refers to the co-occurrence of self- and other-oriented harmful behaviors. A systematic review study by Shafti et al. (2023) provided evidence that dual-harm may not be a distinct clinical entity. Instead, it may emerge from the interaction of intrapersonal and interpersonal risk factors that can also be linked to both self- and other-oriented harmful behaviors. As such, individuals engaging in either self- or other-oriented harmful behaviors may be considered at risk for exhibiting the other (O’Donnell et al., 2015). It is difficult to draw clear conclusions on the nature and extent of dual-harmful behaviors, as studies vary in their operationalization of dual-harm. There is a considerable body of research defining dual-harm as the co-occurrence of homicide and suicide (for an overview, see Panczak et al., 2013), but other harmful behaviors can also be the focus of dual-harm studies. Spaan et al. (2022), for example, measured aggressive behaviors and violent behaviors as other-oriented harm, and NSSI and suicidality as self-oriented harm. Harford and colleagues (2013) measured other-oriented and self-oriented harm with five items (e.g., “ever use a weapon like a stick, knife, or gun in a fight”) and four items (e.g., “felt like wanting to die”), respectively. O’Donnell and colleagues (2015) found that the majority of the studies on combined self-oriented and other-oriented harmful behaviors report a prevalence rate of dual-harm equaling or exceeding 20%. Dual-harm seems more frequent in men than in women, but these findings should be interpreted with caution as only a handful of studies has looked at sex differences in dual-harm (O’Donnell et al., 2015). Importantly, individuals reporting dual-harm have more severe psychopathology and are more likely to die before the age of 35 than individuals who engage in either self- or other-oriented harmful behavior (Harford et al., 2013, 2016; Spaan et al., 2022; Steeg et al., 2019).

While prior work has focused on intrapersonal and interpersonal risk factors of self-, other-oriented, and dual-harmful behaviors (e.g., Harford et al., 2016), still little is known about the role of reactive and regulative temperamental traits in relation to the co-occurrence of harmful behaviors. Previous studies have mainly investigated the influence of temperamental dimensions on other- and self-oriented harmful behaviors separately, without taking dual-harm into account. Temperamental dimensions have been identified as psychological risk factors underlying both types of behaviors (Bijttebier et al., 2009; Santens et al., 2020). Therefore, we aim to investigate how reactive and regulative temperament dimensions differentiate the full spectrum of harmful behaviors, including individuals not engaging in harmful behavior (i.e., the no-harm group), only engaging in self-oriented harmful behavior (i.e., the NSSI-only group), only engaging in other-oriented harmful behavior (i.e., the aggression-only group), and reporting both types of behaviors (i.e., the dual-harm group).

### Temperament

Temperament refers to individual differences in (1) emotional reactivity and (2) self-regulation, which are relatively stable across situations and over time (Rothbart et al., 2000). Reactive temperament has been described in the revised-Reinforcement Sensitivity Theory (r-RST) of Gray and McNaughton (2000). The r-RST comprises three systems: a Behavioral activation system (BAS), a fight-flight-freeze system (FFFS), and a Behavioral inhibition system (BIS). BAS reflects a general approach tendency connected to reward sensitivity, positive affect, and extraversion (Corr, 2008). BAS consists of four empirically validated dimensions: BAS-Reward interest, BAS-Goal-drive persistence, BAS-Reward reactivity, and BAS-Impulsivity. BAS-Reward interest and BAS-Goal-drive persistence both reflect reward desire and are associated with respectively exploration and drive, whereas BAS-Reward reactivity and BAS-Impulsivity are activated in reaction to rewarding stimuli and are associated with respectively responsiveness and non-planning. In addition, FFFS and BIS reflect a general avoidance tendency connected to punishment sensitivity, negative affect, and neuroticism (Corr, 2008). In this study, FFFS refers to the flight-freeze system, excluding the fight-component (see Corr & Cooper, 2016). The flight-freeze system is conceptualized as an avoidance system promoting fleeing or freezing behavior in reaction to a stimulus, depending on the perceived danger of the stimulus. The flight-freeze system is associated with fear and panic (Corr, 2008). BIS functions as a conflict detector and regulator of BAS reactivity and flight-freeze reactivity: A conflict within or between them is followed by an increase in arousal, motivating individuals to resolve the detected conflict. For example, when a person encounters a situation where fleeing may coincide with a desire to stay (flight-freeze system activation vs BAS), BIS detects this conflict and increases arousal to motivate resolution, such as evaluating the level of threat and opting for the safer option. BIS is linked to increased anxiety (Corr, 2008). In terms of sex differences, women tend to report more BAS-Goal-drive persistence, BAS-Reward reactivity, flight-freeze reactivity, and BIS compared to men, and men tend to report more BAS-Reward interest and BAS-Impulsivity compared to women (Dierickx et al., 2020; Ma-Kellams & Wu, 2020).

Regulative temperament, described in terms of Effortful control (EC), is defined as the top-down capacity to moderate the reactivity of BAS, the flight-freeze system, and BIS to elicit adaptive behavioral responses (Derryberry & Rothbart, 1997). EC generally consists of three components: (1) attentional control is the ability to voluntarily focus or shift attention when needed, (2) activation control involves the ability to act even when lacking motivation, and (3) inhibitory control is the ability to voluntarily inhibit behavior (Rothbart & Derryberry, 2013). EC is positively associated with conscientiousness (Nigg, 2006). Studies report no significant sex differences in EC in adults (Busch & Hofer, 2012; Meehan et al., 2013).

### The associations between temperament and harmful behaviors

Several cross-sectional studies have investigated the association between temperament and NSSI. These studies revealed that higher BIS reactivity is related to NSSI engagement (Ammerman et al., 2016; Baetens et al., 2011; Gandhi et al., 2016; Jenkins et al., 2013; Wu et al., 2021; Ying et al., 2016). The findings regarding the association between BAS and NSSI are contradictory (Gandhi et al., 2016; Jenkins et al., 2013; Raemen et al., 2020; Wu et al., 2021; Ying et al., 2016), with some studies finding positive and other studies finding negative or no significant associations with NSSI. Up till now, studies on the relationship between NSSI and flight-freeze-reactivity do not exist. Additionally, low levels of EC have consistently been linked to more NSSI engagement (Baetens et al., 2011; Esposito et al., 2022; Gandhi et al., 2016), even more so in interaction with high BIS (Raemen et al., 2020).

When focusing on the relationship between temperament and aggressive behavior, cross-sectional findings systematically show that BIS is negatively associated with aggressive behavior, whereas BAS, especially BAS-Impulsivity, is positively associated with aggressive behavior (Megías-Robles et al., 2021; Seibert et al., 2010; Smits & Kuppens, 2005; von Collani & Werner, 2005). Until now, research on the relationship between aggressive behavior and flight-freeze-reactivity is lacking. Finally, EC is negatively related to aggressive behavior (Dane & Marini, 2014).

To date, there are no studies focusing on the associations between temperament and engaging in dual-harm (NSSI and aggressive behavior combined). However, it is known that the borderline personality disorder is highly prevalent (70.7%) among individuals engaging in dual-harm compared to the prevalence (11.4%) in the general population (Harford et al., 2019). The prototypic temperamental profile of individuals with borderline personality disorder is characterized by high BIS, high BAS, and low EC (Sleuwaegen et al., 2017).

It is important to note that the aforementioned findings on NSSI, aggressive behaviors and dual-harm are mostly stemming from studies that (1) make use of instruments based on the original-RST instead of the newer and empirically supported r-RST (Corr, 2008), (2) focus on adolescents, students, or adult populations, and (3) consider the relation between temperamental dimensions among either self-oriented harmful behaviors or other-oriented harmful behaviors, but do not include both types of harmful behaviors in one study. By consequence, the similarities and differences considering temperamental traits in emerging adults who engage in no harmful behaviors, in either self- or other-oriented harmful behaviors, or those who engage in a combination of both harmful behaviors, remain unexplored.

### Rationale of the study

To address these gaps in the existing literature, the present study examines which temperamental traits can differentiate four different groups (i.e., a no-harm, NSSI-only, aggression-only, and a dual-harm group) in a pairwise manner, while controlling for age and sex, in a sample of emerging adults.

We hypothesize that the likelihood of engaging in NSSI-only, compared to no-harm or aggression-only, will be positively associated with higher BIS (Ammerman et al., 2016; Baetens et al., 2011; Esposito et al., 2022; Gandhi et al., 2016; Jenkins et al., 2013; Raemen et al., 2020; Wu et al., 2021; Ying et al., 2016). Additionally, we expect that individuals who engage in aggression-only will report lower BIS and higher BAS-Impulsivity than the group with no harmful behaviors or the individuals reporting NSSI-only (Dane & Marini, 2014; Megías-Robles et al., 2021; Seibert et al., 2010; Smits & Kuppens, 2005; von Collani & Werner, 2005). The hypothesis regarding the flight-freeze system is exploratory in nature. Considering dual-harm, the analyses are exploratory, but based on the strong link between dual-harm and borderline personality disorder (Harford et al., 2019), we assume that the odds of engaging in both NSSI and aggressive behavior, compared to no-harm, NSSI-only, or aggression-only, are positively associated with BIS and BAS and negatively with EC (Sleuwaegen et al., 2017).

## Methods

### Participants and procedure

The present study is part of a larger research project focusing on the relationship between self- and other-oriented harmful behaviors and temperament among emerging adults. In total, we collected data from 847 participants. Due to the fact that the present study focuses on NSSI and aggressive behaviors or a combination of both, we excluded participants who did not complete the questionnaires on NSSI and aggressive behavior. The remaining participants were 669 emerging adults aged 18–25 years old (*M*_age_ = 21.48; *SD* = 2.20), of whom 205 (30.64%) identified as men and 464 (69.36%) identified as women. As the data was collected in Belgium, 644 of the 669 participants had the Belgian nationality (96.26%), of which 8 reported a double nationality (1.20% of the total sample), and 25 participants reported a different nationality (3.74%).

A snowball sampling technique was used to collect data from October 2021 until April 2022 during the COVID-19 pandemic. Invitations to participate in an anonymous web-based survey (i.e., informed consent form, sociodemographic items and eight questionnaires) were sent to social organizations (e.g., youth movement clubs, sports clubs, music societies, student societies) to distribute among their Dutch-speaking emerging adult members (18 to 26 years old). Only the questionnaires that are relevant for the present study are described below. The study was approved by the Social and Societal Ethics Committee of KU Leuven under file number G-2021–3870-R2(MAR).

### Materials

The sociodemographic variables that were included are sex (man/woman) and age (in years).

Lifetime NSSI was assessed by means of a single dichotomous (yes/no) item ‘Have you ever engaged in self-injury without an intent to die?’. A definition of NSSI was offered to the participants to clarify that non-suicidal self-injury included harmful behaviors oriented towards the self, such as carving or cutting oneself, but without suicidal intent. The use of a single dichotomous item is common in NSSI research and often leads to a consistent estimation of lifetime NSSI prevalence (Muehlenkamp et al., 2012).

Aggressive behavior was operationalized by the ‘direct aggression’ scale of the Buss-Durkee Hostility Inventory-Dutch (BDHI-D; Lange et al., 2005). The scale consists of 16 items (e.g., “When I really lose my temper, I am capable of slapping someone”) to be rated as true or false (α = .77). A total score ≥7.05 on the direct aggression scale indicates the presence of aggressive behavior (compared to the norm group of the general Dutch population aged 18–40 years old; Lange et al., 2005).

Utilizing lifetime NSSI and direct aggression, we constructed four groups: (1) those who reported neither NSSI nor aggressive behavior (no-harm group), (2) those who reported only NSSI (NSSI-only group), (3) those who reported only aggressive behavior (aggression-only group), and (4) those who reported both NSSI and aggressive behavior (dual-harm group).

Reactive temperament (BAS, flight-freeze system, and BIS) was assessed by means of the Brief-Reinforcement Sensitivity Theory of Personality Questionnaire (B-RST-PQ; Dierickx et al., 2020). The B-RST-PQ consists of 37 items which are rated on a 4-point Likert scale ranging from 1 (Not at all accurate) to 4 (Highly accurate). BAS is split in four BAS-subscales: BAS-Reward interest (5 items; e.g., “I regularly try new activities just to see if I enjoy them”; α = .79), BAS-Goal-drive persistence (5 items; e.g., “I am very persistent in achieving my goals”; α = .85), BAS-Reward reactivity (4 items; e.g., “I find myself reacting strongly to pleasurable things in life”; α = 68) and BAS-Impulsivity (5 items; e.g., “I find myself doing things on the spur of the moment”; α = .73). The flight-freeze system consists of 6 items (e.g., “Looking down from a great height makes me freeze.”; α = .67 in the present study). Finally, BIS consists of 12 items (e.g., “I am often preoccupied with unpleasant thoughts.”; α = .91 in the present study).

Regulative temperament (EC) was assessed by means of the ‘Effortful control’ scale of the Adult Temperament Questionnaire Short Form (ATQ-ECS; Evans & Rothbart, 2007). The ATQ-ECS consists of 19 items (e.g., “I often find it difficult to switch between different tasks” [reverse-coded]); α = .81 in the present study) to be rated on a 7-point Likert scale ranging from 1 (Not at all applicable) to 7 (Completely applicable).

### Analyses

SPSS version 28 was used to analyze the data. A series of logistic regression models with two-sided significance tests were performed with the pairwise group comparisons as dependent variables, temperamental traits as independent variables, and age/sex as control variables. Odds ratios, 95% confidence intervals and Nagelkerke R^2^ are reported. Odds ratios provide insight into the magnitude and direction of associations between predictor variables and the compared groups. An odds ratio higher than 1 indicates higher odds of belonging to the group under investigation compared to the reference group, whereas an odds ratio less than 1 suggests lower odds of belonging to the group under investigation compared to the reference group. Nagelkerke’s R^2^ provides a measure of the model’s overall fit. Given the number of estimated logistic regression models, we conducted a Bonferroni correction to identify strong associations within each model, by dividing the significance level by the number of estimated models (*n* = 6), resulting in a significance level of *p* < .008. The interactions between reactive temperament (BAS, flight-freeze system, and BIS) and regulative temperament (EC) were entered as a second block in each logistic regression model. As they were all found to be statistically non-significant, these were not presented in the manuscript. However, they are added in a supplementary table to the manuscript (Supplementary Materials 1). To further explore the role of significant predictors identified in the results, we will conduct additional ANOVA analyses for each independent variables that emerges as a significant predictor of subgroup membership. Detailed results of these analyses are provided in the supplementary materials (Supplementary Materials 2).

## Results

**Figure 1:**
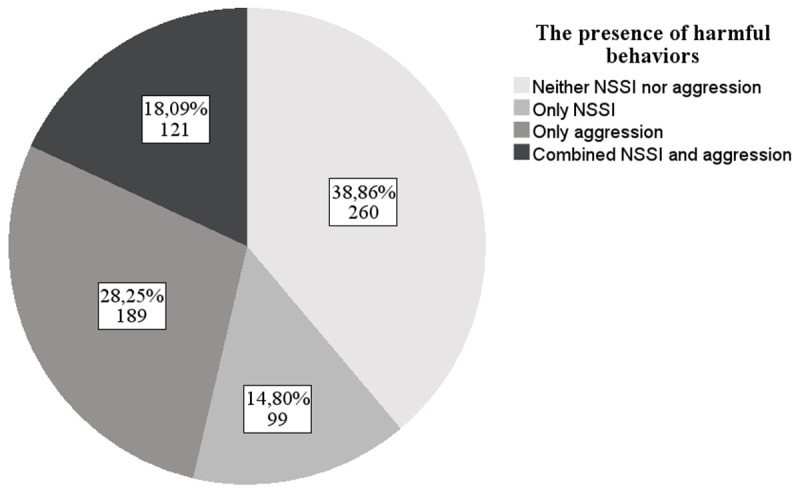
Participants grouped according to reported harmful behaviors. *Note*. NSSI = Non-suicidal self-injury. The pie chart displays the number of participants and percentages.

Lifetime NSSI was estimated at 32.88% (*n* = 220) and aggressive behavior was estimated at 46.34% (*n* = 310). Of all participants, 38.86% (*n* = 260) reported no harm, 14.80% (*n* = 99) reported NSSI-only, 28.25% (*n* = 189) aggression-only, and 18.09% (*n* = 121) reported dual-harm (see Figure 1). Table 1 displays the results of the six logistic regression analyses. The pairwise comparisons are structured as a continuum, ranging from no-harm to single-harm (either self-oriented or other-oriented harm) and dual-harm. This continuum allows us to capture a more nuanced understanding of self-oriented, other-oriented, and dual-harmful behaviors in relation to temperamental traits. Nagelkerke’s R^2^ indicates that the sociodemographic and temperament variables in the model comparisons explain between 16.2% and 37.4% of the variance in group membership.

**Table 1:** Multivariate logistic regression analyses examining reported harmful behaviors.


	COMPARISON 1: NO-HARM (*n* = 260) VERSUS NSSI-ONLY (*n* = 99; REFERENT)	COMPARISON 2: NO-HARM (*n* = 260) VERSUS AGGRESSION-ONLY (*n* = 189; REFERENT)	COMPARISON 3: NO-HARM (*n* = 260) VERSUS DUAL-HARM (*n* = 121; REFERENT)	COMPARISON 4: NSSI-ONLY (*n* = 99) VERSUS AGGRESSION-ONLY (*n* = 189; REFERENT)	COMPARISON 5: NSSI-ONLY (*n* = 99) VERSUS DUAL-HARM (*n* = 121; REFERENT)	COMPARISON 6: AGGRESSION-ONLY (*n* = 189) VERSUS DUAL-HARM (*n* = 121; REFERENT)

	OR (95% CI)	OR (95% CI)	OR (95% CI)	OR (95% CI)	OR (95% CI)	OR (95% CI)

Age	0.95 (0.85–1.07)	0.92 (0.84–1.01)	0.81 (0.81–1.03)	1.00 (0.88–1.13)	1.06 (0.92–1.22)	0.99 (0.88–1.11)

Identifying as man	0.34 (0.17–0.67)**	0.94 (0.59–1.51)	0.65 (0.35–1.20)	2.36 (1.15–4.84)*	1.61 (0.69–3.61)	0.77 (0.42–1.42)

Flight-freeze system	0.73 (0.54–0.99)*	0.96 (0.76–1.22)	0.62 (0.46–0.85)**	1.26 (0.93–1.71)	0.96 (0.68–1.35)	0.76 (0.57–1.01)

Behavioral inhibition system (BIS)	1.82 (1.33–2.50)***	0.78 (0.61–1.00)*	1.70 (1.23–2.36)***	0.42 (0.29–0.59)***	0.88 (0.60–1.29)	1.90 (1.38–2.61)***

Behavioral activation system (BAS)						

Reward interest (RI)	0.94 (0.66–1.35)	0.68 (0.51–0.90)*	0.60 (0.43–0.85)**	0.71 (0.49–1.02)	0.61 (0.42–0.89)**	0.90 (0.65–1.24)

Goal-drive persistence (GDP)	0.71 (0.52–0.97)*	0.93 (0.73–1.19)	1.18 (0.87–1.59)	1.39 (0.99–1.94)	1.65 (1.16–2.35)**	1.22 (0.90–1.66)

Reward reactivity (RR)	0.67 (0.49–0.91)*	0.91 (0.71–1.17)	0.73 (0.53–1.01)	1.17 (0.86–1.59)	0.88 (0.63–1.25)	0.81 (0.60–1.10)

Impulsivity (IMP)	1.37 (0.96–1.96)	2.40 (1.81–3.19)***	2.15 (1.53–3.02)***	1.95 (1.35–2.82)***	1.79 (1.22–2.62)**	0.93 (0.67–1.28)

Effortful control (EC)	1.14 (0.81–1.59)	0.98 (0.74–1.29)	0.61 (0.43–0.87)**	0.76 (0.53–1.09)	0.49 (0.33–0.73)***	0.64 (0.45–0.91)*

Nagelkerke R^2^	0.207	0.162	0.374	0.258	0.243	0.214


*Note*. *p < .05. **p < .01. ***p < .001. Odds ratios significant after applying the Bonferroni correction are boldfaced. OR = Odds Ratio. CI = Confidence Interval. NSSI = non-suicidal self-injury.

### No-harm vs. NSSI-only, aggression-only, and dual-harm

After Bonferroni correction, the odds of belonging to the NSSI-only group over the no-harm group (comparison 1 of Table 1) are positively related to BIS. Individuals who only engage in NSSI show higher levels of BIS reactivity (anxiety) compared to individuals who do not engage in either NSSI nor aggressive behaviors (see also Supplementary Materials 2, for an overview of the mean scores of BIS across subgroups).

The odds of belonging to the aggression-only group over the no-harm group (comparison 2 of Table 1) are positively related to BAS-Impulsivity. This result implies that individuals who engage in aggressive behaviors tend to report higher BAS-Impulsivity (approach reward without planning) compared to individuals who engage in neither NSSI nor aggressive behavior (see also Supplementary Materials 2, for an overview of the mean scores of BAS-Impulsivity across subgroups).

The odds of belonging to the dual-harm group over the no-harm group (comparison 3 of Table 1) are positively associated with BIS reactivity and BAS-Impulsivity. These results indicate that individuals who engage in both NSSI and aggressive behaviors have a tendency to report higher BIS (anxiety) and BAS-Impulsivity (approach reward without planning) compared to those who engage in neither harmful behavior.

### NSSI-only vs. aggression-only and dual-harm

The odds of belonging to the aggression-only group over the NSSI-only group (comparison 4 of Table 1) are positively associated with BAS-Impulsivity and negatively with BIS reactivity. This means that individuals who only engage in aggressive behaviors are more likely to exhibit higher levels of BAS-Impulsivity (approach reward without planning) and lower levels of BIS (anxiety) compared to individuals who only engage in NSSI.

The odds of belonging to the dual-harm group over the NSSI-only group (comparison 5 of Table 1) are negatively associated with EC, implying that individuals who engage in both NSSI and aggressive behaviors report less EC (conscientiousness) than individuals who only engage in NSSI (see also Supplementary Materials 2, for an overview of the mean scores of EC across subgroups).

### Aggression-only vs. dual-harm

Finally, the odds of belonging to the dual-harm group compared to the aggression-only group (comparison 6 of Table 1) are positively related to BIS. This result implies that individuals who engage in both NSSI and aggressive behaviors are more likely to show higher BIS (anxiety) than those who only engage in aggressive behaviors.

## Discussion

The objective of this study was to explore which reactive and regulative temperamental traits can serve as transdiagnostic or unique factors underlying engagement in self-oriented, other-oriented, and dual-harmful behaviors in a sample of emerging adults. The following findings are of particular significance.

### No-harm vs. NSSI-only, aggression-only, and dual-harm

When comparing the no-harm group with individuals who engage in only NSSI, only aggressive behaviors, or a combination of both, the results support earlier findings which related temperamental dimensions against the presence or absence of either harmful behavior.

First, BIS, i.e., high anxiety, has a positive impact on the odds of belonging to the NSSI-only group versus the no-harm group. This finding is in accordance with previous studies that support higher BIS reactivity among individuals engaging in NSSI compared to those without NSSI (e.g., Baetens et al., 2011; Claes et al., 2014; Jenkins et al., 2013; Raemen et al., 2020). These individuals high in BIS often exhibit higher levels of affect-related dysregulation, as found in depression (e.g., Bijttebier et al., 2009; Wu et al., 2021), which may drive individuals to the use of NSSI as an affect-regulatory strategy to alleviate intense negative emotions (Nock & Prinstein, 2004).

BAS-Impulsivity, i.e., a tendency to approach reward without planning, significantly increases the odds of aggressive behavior over no harmful behavior. These results are in line with a considerable body of evidence showing that individuals exhibiting broader impulse control issues show a higher propensity for direct aggressive behavior (Bettencourt et al., 2006; Miller et al., 2012; Parker et al., 2022).

Finally, high BIS and high BAS-Impulsivity increase the odds of engaging in both NSSI and aggressive behaviors over engaging in neither harmful behavior. The profile of high BIS and high BAS-Impulsivity is typically seen in individuals with borderline personality disorder who also report intense emotionality and high disinhibition and impulsivity (Sleuwaegen et al., 2017). The above findings support interventions targeting emotional regulation and impulse control, as found in empirically supported treatments such as dialectical behavior therapy (DBT; Linehan, 2020), to reduce dual-harmful behaviors (Wetterborg et al., 2020). Multiple studies have demonstrated the efficacy of DBT in individuals engaging in NSSI (Chapman et al., 2023) and aggressive behaviors (Ciesinski et al., 2022). Future research should seek to investigate the effectiveness of these interventions to those engaging in dual-harm.

The flight-freeze system does not seem to differentiate between no-harm and the (co-)occurrence of harmful behaviors, i.e., only NSSI, only aggression, or dual-harm. This is an important result as the relationship between the flight-freeze system and harmful behaviors has not been previously explored. While the flight-freeze system and BIS are jointly part of a general avoidance tendency, the results demonstrate that BIS explains more variance in harmful behaviors than the flight-freeze system.

### NSSI-only vs. aggression-only and dual-harm

As far as the authors know, no studies compared temperamental reactivity between an NSSI-only group and a group with aggression-only or dual-harm. The results show that BIS (anxiety) has a negative impact on the odds of aggression-only as opposed NSSI-only, whereas BAS-impulsivity (impulsive action without thinking about the consequences of one’s behavior) positively impacts the odds of engaging in aggression-only above NSSI. These findings support earlier research that elevated BAS and reduced BIS are related to aggressive behaviors (Dane & Marini, 2014; Megías-Robles et al., 2021; Seibert et al., 2010; Smits & Kuppens, 2005; von Collani & Werner, 2005). These findings also align with the profile of psychopathy (Bijttebier et al., 2009; Lykken, 2013; Wallace et al., 2009). Individuals with a weak BIS (primary psychopathy) do not experience sufficient anxiety to inhibit antisocial behaviors, whereas a strong BAS, especially BAS-Impulsivity (secondary psychopathy), drives aggressive behaviors due to the tendency to act without thinking (Bijttebier et al., 2009).

The inverse findings hold for NSSI-only group, compared to the aggression-only group. The NSSI-only group is characterized by high BIS and low BAS-Impulsivity reactivity compared to the aggression-only group. Several prior studies have supported a negative relationship between BAS-Impulsivity and NSSI (Jenkins et al., 2013; Raemen et al., 2020; Wu et al., 2021), but the results so far were inconclusive and based on the original RST instead of r-RST. Elevated BIS and reduced BAS fits the temperamental profile of depression (Bijttebier et al., 2009), which can explain the link between NSSI and depressive symptomatology (e.g., Boyne & Hamza, 2022; Dierickx et al., 2023). Presumably, individuals may resort to NSSI engagement to evoke positive feelings (missing due to low BAS) and/or to reduce their negative affect (related to high BIS; Beevers & Meyer, 2002; Kuan et al., 2024; Nock & Prinstein, 2005).

EC, i.e., conscientiousness, is lower in individuals engaging in dual-harm compared to individuals engaging in NSSI-only. This finding is in line with a studies of Slade et al. (2020), Richmond-Rakerd et al. (2019), and Spaan et al. (2022), which showed that individuals who engage in dual-harm struggle with top-down control (or EC) to regulate reactive emotions when facing distressing situations.

### Aggression-only vs. dual-harm

Studies have not yet examined differences in temperamental reactivity between individuals who engaged exclusively in aggressive behaviors and those who engage in both aggressive behaviors and NSSI (dual-harm). The results indicate that high BIS, i.e., anxiety, increases the likelihood of engaging in dual-harm as opposed to only engaging in aggressive behaviors. These findings highlight the role of BIS in dual-harm, where elevated anxiety may contribute to a repetitive cycle of harmful actions. In contrast, the aggression-only group tends to report lower sensitivity to anxiety, suggesting the behavior is more impulsive rather than driven by internalized distress. These findings support the cognitive-emotional model of dual-harm (Shafti et al., 2021) which suggests that individuals prone to emotional instability, interpersonal difficulties, and maladaptive coping – factors all positively correlated with BIS (Corr, 2008) – may be particularly susceptible to dual-harm.

### Synthesis

The discussed findings have both theoretical and clinical implications. In terms of theoretical implications, this study underscores the importance of administering the r-RST (Corr, 2008) above the original RST. The r-RST seems to offer more nuance. For example, BIS and the Flight-freeze system show two different dynamics even though they are both part of a general avoidance tendency system. BAS-Impulsivity seems to play a more important role in differentiating engagement in harmful behaviors than the other BAS-subscales.

Clinically, the findings in this study support the need for tailored interventions for individuals engaging in different harmful behaviors. Evidence-based treatments of NSSI and aggression, such as Dialectical Behavioral Therapy (DBT; Linehan, 2020) or Cognitive-Behavioral Therapy (Lee & DiGiuseppe, 2018), often include training strategies to replace harmful behaviors with more adaptive behavioral strategies. Based on the findings in this manuscript which show that BIS, BAS-Impulsivity, and EC differentiate between no-harm, NSSI-only, aggression-only, and dual-harm, we need to encourage individuals who engage in NSSI to develop emotion-regulating behaviors that are not harmful (Prada et al., 2018), whereas for individuals who engage in aggression, we recommend focusing on impulse-regulation skills (Schaich et al., 2021). In the case of dual-harm, both emotion and impulse regulation skills are needed, which is the case in programs such as DBT.

Although the present study offers valuable insights as one of the few studies currently available that considers temperamental dimensions underlying both self-oriented and other-oriented forms of harmful behavior, several limitations warrant consideration. The study used cross-sectional data from a community sample collected through snowball sampling. As we collected only limited sociodemographic information (age, sex, and nationality), our ability to assess the relevance of these findings across different demographic groups is restricted. Although this recruitment method is practical for reaching individuals who engage in NSSI and/or aggressive behaviors, it may limit the generalizability of the findings. Future research could address this limitation by employing randomized sampling methods. Replicating the study in clinical populations would also provide a more comprehensive understanding of these behaviors and allow for exploration of the potential benefits of therapeutic interventions on self-oriented, other-oriented, or dual-harm.

Additionally, longitudinal research is needed to examine developmental trajectories and ascertain whether engaging in NSSI, aggressive behavior and dual-harm are more than merely associated with temperamental traits. Future studies should explore whether reactive and regulative temperament differentially predict NSSI, aggression, and dual-harm. Longitudinal research can also contribute to our understanding of engagement in NSSI and aggressive behavior as possible risks for more adverse outcomes over time. Moreover, in the present study, we included a combination of NSSI and aggressive behavior to operationalize dual-harmful behaviors. However, there is no consensus on which types of harmful behaviors should be included to constitute dual-harm, or on whether there should be a cutoff to establish recency or severity of the behaviors included. Future studies should examine a broader range of harmful behaviors in relation to each other, also considering their diverse characteristics, such as behavioral expressions, persistence, and thoughts related to the harmful behaviors. In that perspective, the work of Bresin (2020) offers a meaningful overview of diverse types of harmful behaviors (e.g., aggression, NSSI, as well as substance use, binge eating and gambling).

Finally, there is a close relationship between intrapersonal functioning (e.g., temperament, emotion regulation) and interpersonal functioning (e.g., parental criticism, abuse), which are accredited in conceptual models of both NSSI (e.g., Nock, 2009; Selby & Joiner, 2009) and aggressive behaviors (Anderson & Bushman, 2002). The present study focused on intrapersonal factors, i.e., temperamental dimensions, and did not include other factors that may have mediated or moderated the relation between temperament and harmful behaviors. Building upon the present study, future research could examine how interpersonal factors, as well as interactions between intrapersonal and interpersonal factors might play a role in the association between temperament and harmful behaviors.

## Conclusion

In summary, the findings of this study reveal that reactive and regulative temperament are important transdiagnostic factors underlying engagement in self-oriented, other-oriented, and dual-harmful behaviors. Specifically, an elevated BIS and a decreased BAS-Impulsivity are linked to a greater likelihood of engaging in NSSI, as opposed to reporting no harmful behaviors or only aggressive behaviors. Conversely, a decreased BIS and an elevated BAS-Impulsivity are linked with a propensity for engaging in aggressive behaviors, compared to reporting no harmful behaviors or only NSSI. Individuals exhibiting dual-harmful behaviors demonstrate a deficit in EC, indicating lower levels of self-regulation, compared to individuals engaging only NSSI, and high BIS and BAS-Impulsivity compared to those engaging only aggressive behaviors or in neither NSSI nor aggressive behaviors. These differential associations highlight the nuanced interplay between temperamental traits and specific manifestations of harmful behaviors among emerging adults, which should be considered in future research and clinical practice.

## Reproducibility

The data supporting the findings of this study are available upon reasonable request. Please contact the first author for access to the data.

## Additional Files

The additional files for this article can be found as follows:

10.5334/pb.1327.s1 Supplementary Material 1. Table S1.

10.5334/pb.1327.s2 Supplementary Material 2. Tables S2 and S3.
