# Supplementary material for: The Associations Between Temperament and Self-Oriented, Other-Oriented, and Dual-Harmful Behaviors in Emerging Adults
Source: Psychol Belg. 2024 Dec 30;64(1):201–13. doi: 10.5334/pb.1327 (PMC11697618; doi:10.5334/pb.1327)
Supplement: Supplementary Material 1. — Table S1. [file pb-64-1-1327-s1.pdf]

Supplementary materials 1

Table S1.

Multivariate logistic regression analyses examining reported harmful behaviors.

	No-harm (*n*=260) versus NSSI-only (*n*=99)	No-harm (*n*=260) versus aggression-only (*n*=189)	No-harm (*n*=260) versus dual-harm (*n*=121)	NSSI-only (*n*=99) versus aggression-only (*n*=189)	NSSI-only (*n*=99) versus dual-harm (*n*=121)	Aggression-only (*n*=189) versus dual-harm (*n*=121)
	OR (95% CI)	OR (95% CI)	OR (95% CI)	OR (95% CI)	OR (95% CI)	OR (95% CI)
Age	0.95 (0.85-1.07)	**0.91 (0.83-1.00)***	0.93 (0.82-1.05)	0.98 (0.87-1.12)	1.08 (0.94-1.24)	1.00 (0.89-1.13)
Identifying as man	**0.33 (0.17-0.67)****	0.99 (0.61-1.60)	0.66 (0.35-1.24)	**2.45 (1.16-5.15)***	1.58 (0.68-3.71)	0.79 (0.43-1.46)
Flight-freeze system	**0.73 (0.54-0.99)***	0.97 (0.76-1.23)	**0.66 (0.47-0.91)***	1.29 (0.94-1.79)	1.01 (0.70-1.46)	0.79 (0.58-1.07)
Behavioral inhibition system (BIS)	**1.77 (1.29-2.44)*****	**0.76 (0.59-0.97)***	**1.79 (1.27-2.53)*****	**0.40 (0.28-0.58)*****	0.87 (0.59-1.29)	**2.07 (1.47-2.90)*****
Behavioral activation system (BAS)						
Reward interest (RI)	0.95 (0.66-1.37)	**0.67 (0.50-0.88)****	**0.62 (0.43-0.89)****	0.72 (0.49-1.06)	**0.65 (0.44-0.97)***	0.82 (0.57-1.19)
Goal-drive persistence (GDP)	**0.69 (0.50-0.94)***	0.94 (0.73-1.21)	1.27 (0.93-1.74)	**1.46 (1.03-2.08)***	**1.81 (1.25-2.62)****	1.28 (0.91-1.80)
Reward reactivity (RR)	**0.68 (0.50-0.93)***	0.90 (0.70-1.15)	0.72 (0.51-1.02)	1.22 (0.88-1.70)	0.93 (0.65-1.34)	0.83 (0.60-1.17)
Impulsivity (IMP)	1.34 (0.93-1.92)	**2.48 (1.86-3.32)*****	**2.23 (1.55-3.20)*****	**2.00 (1.37-2.93)*****	**1.69 (1.13-2.54)***	0.93 (0.65-1.34)
Effortful control (EC)	1.07 (0.74-1.55)	0.94 (0.70-1.26)	**0.54 (0.37-0.79)****	0.84 (0.57-1.24)	**0.50 (0.31-0.80)****	**0.66 (0.45-0.96)***
Flight-freeze system*EC	1.19 (0.88-1.62)	1.25 (1.00-1.58)	1.26 (0.94-1.70)	0.93 (0.66-1.31)	1.05 (0.72-1.53)	1.11 (0.83-1.47)
BIS*EC	1.19 (0.84-1.69)	0.94 (0.73-1.20)	1.35 (0.95-1.90)	0.84 (0.58-1.23)	1.15 (0.75-1.75)	1.25 (0.91-1.70)
BAS-RI*EC	1.00 (0.66-1.52)	0.98 (0.75-1.28)	1.07 (0.74-1.55)	1.05 (0.70-1.60)	1.15 (0.75-1.76)	0.90 (0.64-1.25)
BAS-GDP*EC	0.87 (0.63-1.21)	1.28 (1.00-1.64)	1.27 (0.93-1.73)	1.36 (0.98-1.89)	1.23 (0.86-1.76)	1.10 (0.81-1.48)
BAS-RR*EC	0.75 (0.54-1.03)	0.97 (0.76-1.24)	1.01 (0.72-1.42)	1.22 (0.88-1.70)	1.22 (0.82-1.82)	1.10 (0.80-1.50)
BAS-IMP*EC	1.22 (0.84-1.77)	1.03 (0.78-1.35)	1.03 (0.72-1.47)	1.01 (0.65-1.55)	0.82 (0.52-1.29)	0.92 (0.66-1.28)
Nagelkerke R^2	0.239	0.185	0.401	0.307	0.275	0.232

Note. The findings in this table represent the second block of a series of hierarchical logistic regression models. The first block of these models, without interaction terms between reactive and regulative temperament, are included in Table 1 of the manuscript.

$^{*}p < .05$. $^{**}p < .01$. $^{***}p < .001$. Significant odds ratios are made boldface. OR = Odds Ratio. CI = Confidence Interval.
