# Supplementary material for: The Associations Between Temperament and Self-Oriented, Other-Oriented, and Dual-Harmful Behaviors in Emerging Adults
Source: Psychol Belg. 2024 Dec 30;64(1):201–13. doi: 10.5334/pb.1327 (PMC11697618; doi:10.5334/pb.1327)
Supplement: Supplementary Material 2. — Tables S2 and S3. [file pb-64-1-1327-s2.pdf]

Supplementary materials 2

Table S2 presents the descriptive statistics for the mean scores of the Behavioral Inhibition System (BIS), the Behavioral Activation System (BAS)-Impulsivity, and Effortful Control (EC) for the different subgroups: no-harm, NSSI-only, aggression-only, and dual-harm. Table S3 summarizes the one-way ANOVA results for mean scores on BIS, BAS-Impulsivity, and EC across the four comparison groups. Post hoc comparisons using the Bonferroni correction were conducted to identify specific group differences where significant effects were found.

Post-hoc comparisons using the Bonferroni correction revealed a statistically significant difference in ***BIS scores*** between the no-harm group (*M* = 30.58; *SD* = 7.96) and the NSSI-only group (*M* = 35.42; *SD* = 7.06) and also the dual-harm group (*M* = 36.93; *SD* = 7.37), with NSSI-only group and the dual-harm group demonstrating significantly higher BIS scores than the no-harm group ($p < .001$ for both comparisons). The difference between the no-harm group and the aggression-only group was nonsignificant ($p = 1.000$). In addition, a significant difference was found for the mean BIS score of the NSSI-only group (*M* = 35.42; *SD* = 7.06) and the aggression-only group (*M* = 30.87; *SD* = 8.22), showing significantly higher BIS scores among the NSSI-only group compared to the aggression-only group ($p < .001$). The difference between the NSSI-only group and the dual-harm group was nonsignificant ($p = .937$). Finally, the aggression-only group (*M* = 30.87; *SD* = 8.22) reported significantly lower BIS scores than the dual-harm group (*M* = 36.93; *SD* = 7.37, $p < .001$).

Considering differences in ***BAS-Impulsivity scores***, we found that the no-harm group (*M* = 8.95; *SD* = 2.95) reported significantly lower BAS-Impulsivity scores than the aggression-only group (*M* = 10.85; *SD* = 3.20, p < .001) and the dual-harm group (*M* = 11.51; *SD* = 3.69, $p < .001$). The difference between the no-harm group and the NSSI-only group was nonsignificant ($p = 1.000$). In addition, the NSSI-only group (*M* = 9.25; *SD* = 3.36) demonstrated significantly lower scores on BAS-Impulsivity than the aggression-only group (*M* = 10.85; *SD* = 3.20, $p < .001$) and the dual-harm group (*M* = 11.51; *SD* = 3.69, $p < .001$). The difference between the aggression-only group and the dual-harm group was nonsignificant ($p = 475$).

Considering mean ***EC scores***, we found that the no-harm group (*M* = 4.39; *SD* = 0.76) reported significantly higher EC scores than the aggression-only group (*M* = 4.15; *SD* = 0.81, *p* = .007) and the dual-harm group (*M* = 3.71; *SD* = 0.79, *p* < .001). The difference between the no-harm group and the NSSI-only group was nonsignificant (*p* = .427). In addition, the NSSI-only group (*M* = 4.22; *SD* = 0.76) demonstrated significantly higher scores on EC than the dual-harm group (*M* = 3.71; *SD* = 0.79, *p* < .001). The difference between the NSSI-only group and the aggression-only group was nonsignificant (*p* = 1.000). Finally, the aggression-only group (*M* = 4.15; *SD* = 0.81) scored significantly higher than the dual-harm group (*M* = 3.71; *SD* = 0.79, *p* < .001).

Table S2. Descriptive statistics of BIS, BAS-Impulsivity, and EC for the subgroups differentiating harmful behavior engagement.

	Subgroup	*n*	*Mean*	*SD*
Behavioral Inhibition System (BIS)	No-harm group	260	30.58	7.96
	NSSI-only group	99	35.42	7.06
	Aggression-only group	189	30.87	8.22
	Dual-harm group	121	36.93	7.37
	**Total sample**	**669**	**32.53**	**8.22**
Behavioral Activation System (BAS)-Impulsivity	No-harm group	260	8.95	2.95
	NSSI-only group	99	9.25	3.36
	Aggression-only group	189	10.85	3.20
	Dual-harm group	121	11.51	3.69
	**Total sample**	**669**	**10.00**	**3.39**
Effortful Control (EC)	No-harm group	260	4.39	0.76
	NSSI-only group	99	4.22	0.76
	Aggression-only group	189	4.15	0.81
	Dual-harm group	121	3.71	0.79
	**Total sample**	**669**	**4.17**	**0.81**

Note. NSSI = Non-suicidal self-injury.

Table S3. One-way ANOVA comparing the subgroups differentiating harmful behavior engagement.

		Sum of squares	*Df*	*Mean square*	*F*	*p*
Behavioral Inhibition System (BIS)	Between groups	4670.08	3	1556.69	25.55	< .001
	Within groups	40510.60	665	60.92		
	Total	45180.68	668			
Behavioral Activation System (BAS)-Impulsivity	Between groups	753.78	3	251.26	24.15	< .001
	Within groups	6920.22	665	10.41		
	Total	7673.99	668			
Effortful Control (EC)	Between groups	38.10	3	12.70	20.91	< .001
	Within groups	404.04	665	.61		
	Total	442.14	668			

Note.
